# The Individual Impact of Machine Perfusion on Liver and Kidney on Donor Expansion in Simultaneous Liver and Kidney Transplantation

**DOI:** 10.3389/ti.2025.14807

**Published:** 2025-09-09

**Authors:** Rikako Oki, Ingrid Rocha, Saleh Al-Juburi, Luckshi Rajendran, Emily Kerby, Adhnan Mohamed, Abbas Al-Kurd, Ahmed Nassar, Dean Y. Kim, Atsushi Yoshida, Marwan Abouljoud, Shunji Nagai

**Affiliations:** ^1^ Transplant Institute, Henry Ford Hospital, Detroit, MI, United States; ^2^ Division of Transplant and Hepatobiliary Surgery, Detroit, MI, United States

**Keywords:** donation after circulatory death, donor expansion, kidney donor profile index, machine perfusion, simultaneous liver and kidney transplantation

## Abstract

Machine perfusion (MP) use for both organs can increase organ usage in simultaneous liver and kidney transplantation (SLKT). We analyzed 6,956 SLKT performed between 2015 and 2024 using the United Network for Organ Sharing database. The primary outcomes were the 1-year graft survival for kidney and liver. Donor types and MP use for liver and/or kidney were captured and associations with outcomes were evaluated. SLKT from Donation after circulatory death donors (DCD) increased from 4.5% in 2015 to 16% in 2023. The median Kidney Donor Profile Index (KDPI) has increased from 23% in 2015 to 28% in 2023. MP use for kidney and liver also increased from 21% to 51% and 0%–17%, respectively. KDPI >85% was an independent risk factor of 1-year kidney graft failure in the no kidney MP group [HR 2.03, 95% CI 1.20–3.44, p = 0.009], but not in the kidney MP group. DCD was found to be an independent risk factor of 1-year liver graft failure in the no liver MP group [HR 1.56, 95% CI 1.19–2.03, p = 0.001], but not in the liver MP group. MP for both organs may contribute to expanding the donor pool for SLKT without compromising post-transplant outcomes.

## Graphical Abstract

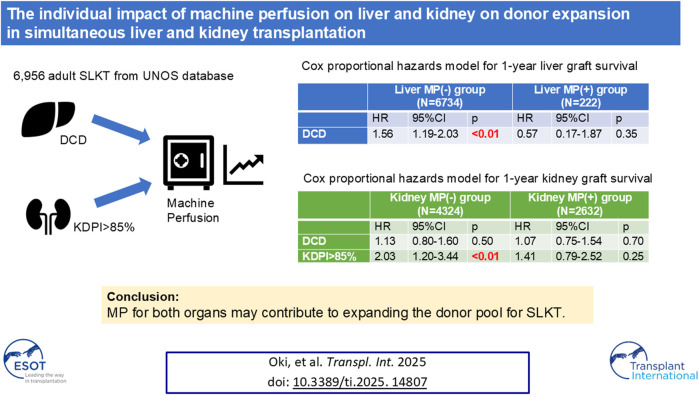

## Introduction

The high demand for organs in both kidney and liver transplantation along with efforts to expand the donor pool has resulted in the increased adoption of machine perfusion (MP) technologies in recent years. Several MP technologies to optimize organ preservation, such as hypothermic machine perfusion (HMP) or normothermic machine perfusion (NMP) have been developed [1].

In liver transplantation, MP could potentially enable the use of lower-quality livers that were not suitable for transplantation before, including donation after circulatory death donors (DCD), older donors, organs with longer cold and warm ischemia time and livers with macrosteatosis [2, 3]. In kidney transplantation, a meta-analysis of 16 studies demonstrated significantly lower DGF and PNF rates in the HMP group compared to those of static cold storage despite having a longer cold ischemia time (CIT) [4]. Furthermore, it has been reported that HMP improved DGF compared with standard cold storage in DCD, particularly in kidneys with a Kidney Donor Profile Index (KDPI) greater than 85%, which are at a higher risk for graft failure [5, 6].

The increase in nationally performed DCD simultaneous liver-kidney transplantations (SLKT) has been observed [7]. Nunez-Nateras et al compared outcomes of donation after brain death (DBD) and DCD in SLKT, and reported similar kidney DGF rates, similar 1-year patient survival (96.7% vs. 95.4% in DCD and DBD), similar 1-year liver allograft survival (93.3% vs. 93.1%) and similar 1-year kidney allograft survival (93.3% vs. 93.1%) [7]. However, MP was not incorporated in this study [7]. The use of MP is also increasing in SLKT, accounting for 1 in 4 kidney allografts since 2017 [8]. Chang et al reported that MP was associated with a reduction in DGF (adjusted Odds Ratio 0.74), but it did not significantly affect PNF. MP were used more often in DCD organs (7.9% vs. 4.5%, p < 0.01) [8]. Given these findings, MP use for both organs in SLKT can potentially increase organ usage from medically complex donors such as those with KDPI >85%, or DCD, without compromising outcomes, but there are few reports which have investigated the individual impact of MP for each organ on donor expansion in SLKT. Thus, the aim of this study was to evaluate 1) the temporal change of donor type and MP use for kidney or liver and 2) compare whether medically complex donors, such as those with KDPI >85% or DCD, pose a risk factor for 1-year graft survival between the use of MP and without MP for each organ in SLKT.

## Materials and Methods

### Patients and Data Collection

This was a retrospective cohort study using the United Network for Organ Sharing (UNOS) database. We identified all adult (≥18 years) recipients of deceased-donor SLKT performed between January 2015, and March 2024. Recipient characteristics (age, gender, race, kidney/liver disease etiology, history of diabetes, body mass index (BMI), Model for End-Stage Liver Disease (MELD) score), severity of liver disease at transplantation (ascites, hepatic encephalopathy, serum albumin (Alb), bilirubin (Bil), international normalized ratio (INR), sodium (Na) at transplantation), severity of renal disease at transplantation (on dialysis or not, creatinine (Cre), estimated glomerular filtration fate (eGFR) at transplantation), and donor characteristics (age, gender, BMI, terminal serum creatinine, donor type, Kidney Donor Profile Index (KDPI)) were obtained from UNOS data. Primary liver disease etiology was reviewed and divided into five groups, alcohol-related liver disease (ALD), nonalcoholic steatohepatitis (NASH), hepatitis C virus infection (HCV), biliary diseases, and others. Primary kidney disease etiology was reviewed and divided into six groups, hepatorenal syndrome, diabetic nephropathy, nephrosclerosis, glomerular nephritis (GN), polycystic kidney disease (PKD) and others. Since the detailed information regarding MP protocols used was not available, it should be noted that MP includes HMP and NMP, which cannot be distinguished in this study. Normothermic regional perfusion (NRP) might have been used but there is not data for it in UNOS. In addition, back-to-base MP might not be captured in UNOS data. Postoperative variable included DGF for kidney, primary non-function (PNF), and re-transplantation. DGF was defined as the requirement for dialysis during the first 7 days following SLKT [9], while PNF for kidney was defined as graft failure of the kidney occurring within 90 days post-transplant [10]. PNF for liver was defined as liver function incompatible with life, requiring retransplantation or resulting in death within 7 days of surgery [11].

This study used the Standard Transplant Analysis and Research file provided by the Organ Procurement and Transplantation Network (OPTN)/UNOS in which all individually identifiable information is encrypted. Henry Ford Institutional Review Board (IRB) exempted IRB approval to conduct this study using this database.

### Outcomes

The primary outcomes included the 1-year graft survival for kidney and liver. “Graft failure” refers to graft failure from any cause, including death and retransplant. For kidney failure, this also includes return to maintenance dialysis. “Graft survival” similarly refers to the absence of all-cause graft failure by the definition of OPTN^1^. First, patients were classified into two groups; kidney MP group (those who used MP for kidney) and no kidney MP group (those who did not use MP for kidney). We evaluated whether KDPI >85% or DCD were identified as a risk factor for 1-year kidney graft failure in kidney MP group and no kidney MP group, respectively. The risks were adjusted for recipient factors such as age [12], gender [13], BMI [14], race [15], diabetes mellitus [16] and race of donor [15] which have been reported to be associated with kidney graft failure in SLKT or kidney only transplantation. Donor factors such as age, BMI, and cause of death which were part of KDPI were not included as adjustment covariables.

Second, patients were classified into two groups; liver MP group (those who used MP for liver) and no liver MP group (those who did not use MP for liver). Similarly, we investigated whether DCD was related to 1-year liver graft failure in liver MP group and no liver MP group, respectively. The risks were adjusted for recipient factors such as age, gender [17], BMI [18], diabetes mellitus [19], race [20], MELD score [21] and donor factors such as age [22], gender [23], BMI [24], race [25], cause of death [25] which have been reported to be associated with liver graft failure/mortality in liver only transplantation.

Third, we also examined the impact of MP on DGF in higher KDPI groups, using KDPI cutoffs of 35, 60, and 85. The risks were adjusted for recipient’s age, gender, BMI, race, diabetes mellitus, donor’s age, gender, BMI, race, and DCD.

Finally, we divided the patients into four group; group1: patients who did not use MP for both kidney and liver, group2: patients who used MP for only kidney, group3: patients who used MP for only liver, group4: patients who used MP for both kidney and liver, and compared the graft survival between the four groups.

### Statistical Analysis

All statistical analyses were conducted using software (SPSS^®^, Version <27.0.1>; IBM Corp., Armonk, NY, United States). Continuous data were expressed as median (interquartile range). Student t-tests or Mann–Whitney U-tests were used to compare continuous variables. The chi-square test or Fisher’s exact test was used to compare the categorical variables. Univariable and multivariable Cox regression analyses were performed to examine significant factors associated with 1-year graft failure. Multivariable logistic regression analyses were performed to examine significant factors associated with DGF for kidney. The Kaplan-Meier method and log-rank test were used to compare differences in 1-year graft survival between four groups divided based on the presence of MP for kidney or liver. *P-values* less than 0.05 were inferred as significant.

## Results

### Trend of SLKT

**FIGURE 1:**
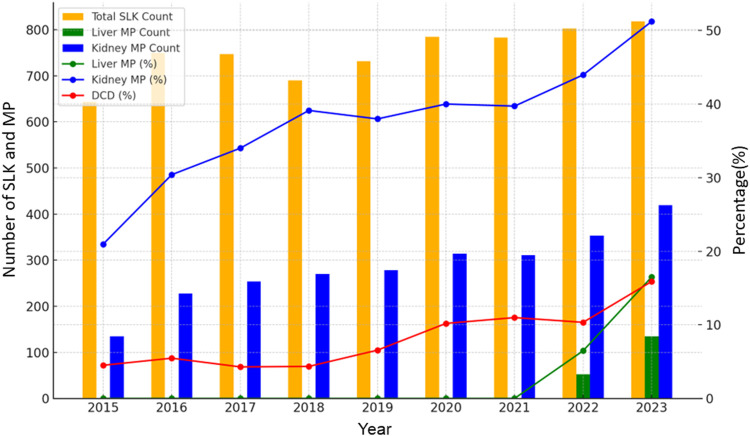
Trends in DCD rate and MP use in SLKT from 2015 to 2023. The number of SLKT transplants showed a steady increasing trend with a slight fluctuation between 2015 and 2023. MP use for kidney and liver also increased from 21% to 51% and 0%–17%, respectively.

**FIGURE 2:**
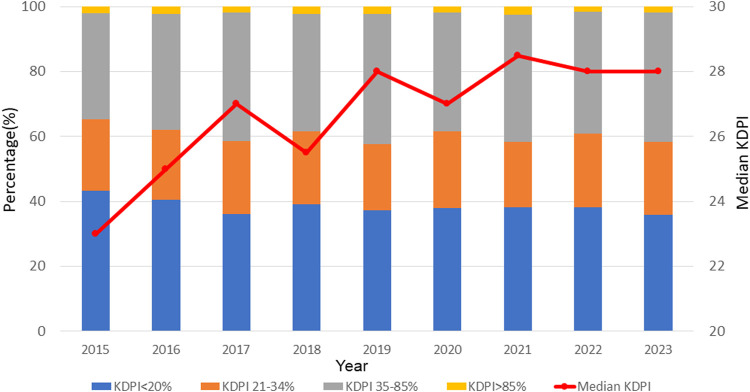
Trends in KDPI in SLKT from 2015 to 2023. Although the ratio of KDPI >85% did not significantly change from 2015 to 2023, the median KDPI has shown an increasing trend from 23 in 2015 to 28 in 2023.

In total, 6,956 adult SLKT were performed during the study period. Between 2015 and 2023, the number of SLKT transplants showed a steady increasing trend with a slight fluctuation, reaching 800 cases in 2022 (Figure 1). SLKT from DCD increased from 4.5% in 2015 to 16% in 2023. MP use for kidney and liver also increased from 21% to 51% and 0%–17%, respectively between 2015 and 2023. Figure 2 demonstrates the changes in KDPI, represented by KDPI categories and the median KDPI. Although the ratio of KDPI >85% did not significantly change from 2015 to 2023, the median KDPI has shown an increasing trend from 23 in 2015 to 28 in 2023 (Figure 2).

### Characteristics of Study Participants

Supplementary Table S1 presents the background characteristics. The average patient age was 59.0 years, of which 59.4% were male. The average donor age was 34.0 years. SLKT from DCD donors accounted for 9.2% of the total. Donor kidneys with a KDPI <20% were the most frequently used, while kidneys with a KDPI >85% accounted for 2.0% of the total. Overall, 37.8% of kidney allografts were placed on MP (N = 2,632) and 3.2% of liver allografts were placed on MP (N = 222).

### Comparison of Patient’ Characteristics According to MP for Kidney or Liver

The comparison of patients’ characteristics between kidney MP group and no kidney MP group is shown in Table 1. Kidney allografts subjected to MP were more frequently DCD (12.2% vs. 7.2%, p < 0.001). Although no statistical difference was found in KDPI category, donors were older in kidney MP group. (median 36.0 vs. 33.0 years, p < 0.001). The severity of kidney disease, as indicated by hemodialysis status, serum creatinine level, and eGFR at the time of transplantation, showed no statistically significant differences (Table 1).

**TABLE 1:** Comparison of background characteristics according to the presence of machine perfusion for kidney or liver allograft.

Recipient Variables	Kidney			Liver		
	No Machine Perfusion (N = 4,324)	Machine Perfusion (N = 2,632)	*p*-value	No Machine Perfusion (N = 6,734)	Machine Perfusion (N = 222)	*p*-value
Age (y.o.)	59.0 (51.0, 64.0)	58.0 (50.0, 64.0)	0.886	59.0 (50.0, 64.0)	60.0 (53.0, 65.0)	0.008
Male (%)	2,578 (59.6)	1,555 (59.1)	0.669	3,996 (59.3)	137 (61.7)	0.488
Race/ethnicity (%)			<0.001			0.131
White	2,647 (61.2)	1743 (66.2)		4,246 (63.1)	144 (64.9)	
Black	553 (12.8)	287 (10.9)		824 (12.2)	16 (7.2)	
Hispanic	840 (19.4)	454 (17.2)		1,248 (18.5)	46 (20.7)	
Asian	207 (4.8)	96 (3.6)		290 (4.3)	13 (5.9)	
Other	77 (1.8)	52 (2.0)		126 (1.9)	3 (1.4)	
BMI (kg/m^2^)	27.4 (23.7, 32.0)	27.6 (24.2, 32.2)	0.014	27.5 (23.9, 32.1)	27.6 (23.8, 32.2)	0.729
Diabetes (%)	1900 (44.0)	1,086 (41.3)	0.028	2,884 (42.9)	102 (45.9)	0.371
Etiology of liver disease (%)			0.013			<0.001
NASH	978 (23.9)	659 (25.2)		1,575 (24.2)	62 (27.9)	
Alcohol	983 (24.0)	615 (23.5)		1,576 (24.3)	22 (9.9)	
HCV	551 (13.4)	281 (10.7)		816 (12.6)	16 (7.2)	
Biliary diseases	152 (3.7)	97 (3.7)		241 (3.7)	8 (3.6)	
Other	1,433 (35.0)	968 (36.9)		2,287 (35.2)	114 (51.4)	
MELD score	29.0 (23.0, 35.0)	28.0 (23.0, 34.0)	0.369	29.0 (23.0, 35.0)	27.0 (21.3, 32.0)	<0.001
Encephalopathy (%)			0.018			0.037
Grade I	1,376 (33.4)	798 (30.4)		2,119 (32.5)	55 (24.8)	
Grade II	2,176 (52.8)	1,422 (54.2)		3,470 (53.2)	128 (57.7)	
Grade III	567 (13.8)	404 (15.4)		932 (14.3)	39 (17.6)	
Ascites (%)	1820 (44.2)	1,236 (47.1)	0.018	2,953 (45.3)	103 (46.4)	0.784
Albumin (g/dL)	3.2 (2.7, 3.7)	3.1 (2.6, 3.6)	0.001	3.2 (2.7, 3.7)	3.2 (2.6, 3.6)	0.399
Sodium (mmol/L)	136.0 (133.0, 139.0)	136.0 (133.0, 139.0)	0.103	136.0 (133.0, 139.0)	136.0 (133.0, 139.0)	0.450
INR	1.6 (1.2, 2.1)	1.5 (1.2, 2.0)	0.135	1.6 (1.2, 2.0)	1.5 (1.2, 1.9)	0.007
Total bilirubin (g/dL)	2.5 (0.9, 7.1)	2.3 (1.0, 6.4)	0.184	2.4 (0.9, 7.0)	1.8 (0.8, 4.8)	0.005
Etiology of kidney disease (%)			<0.001			0.006
Hepatorenal	1701 (39.3)	1,131 (43.0)		2,714 (40.3)	118 (53.2)	
Diabetes	869 (20.1)	492 (18.7)		1,328 (19.7)	33 (14.9)	
PKD	226 (5.2)	175 (6.6)		390 (5.8)	11 (5.0)	
Hypertension	242 (5.6)	106 (4.0)		342 (5.1)	6 (2.7)	
Glomerulonephritis	168 (3.9)	100 (3.8)		259 (3.8)	9 (4.1)	
Other	1,118 (25.9)	628 (23.9)		1701 (25.3)	45 (20.3)	
Hemodialysis (%)	2,810 (68.4)	1792 (68.6)	0.914	4,462 (68.6)	140 (63.3)	0.105
Creatinine (mg/dL)	3.5 (2.3, 5.1)	3.4 (2.3, 5.0)	0.604	3.4 (2.3, 5.1)	3.2 (2.0, 4.7)	0.064
eGFR (mL/min/1.73 m^2^)	17.0 (11.0, 27.3)	17.0 (11.0, 27.4)	0.804	17.0 (11.0, 27.3)	17.6 (11.7, 30.7)	0.126
Donor Variables						
Age (y.o.)	33.0 (24.0, 44.0)	36.0 (27.0, 46.0)	<0.001	34.0 (25.0, 45.0)	38.5 (29.0, 47.0)	<0.001
Male (%)	1,692 (39.1)	1,002 (38.1)	0.388	2,613 (38.8)	81 (36.5)	0.529
Race/ethnicity (%)			0.002			0.052
White	2,737 (63.3)	1795 (68.2)		4,370 (64.9)	162 (73.0)	
Black	663 (15.3)	355 (13.5)		999 (14.8)	19 (8.6)	
Hispanic	754 (17.4)	390 (14.8)		1,113 (16.5)	31 (14.0)	
Asian	108 (2.5)	57 (2.2)		158 (2.3)	7 (3.2)	
Other	62 (1.4)	35 (1.3)		94 (1.4)	3 (1.4)	
BMI(kg/m^2^)	26.2 (23.0, 30.4)	26.7 (23.4, 30.7)	0.001	26.3 (23.1, 30.4)	28.6 (24.7, 32.4)	<0.001
Cause of death (%)			0.438			0.091
anoxia	1868 (43.2)	1,162 (44.1)		2,919 (43.3)	111 (50.0)	
cerebrovascular accident	880 (20.4)	498 (18.9)		1,338 (19.9)	40 (18.0)	
trauma	1,447 (33.5)	884 (33.6)		2,270 (33.7)	61 (27.5)	
Creatinine (mg/dL)	0.90 (0.70, 1.20)	0.88 (0.67, 1.19)	0.003	0.90 (0.70, 1.20)	0.73 (0.60, 1.04)	<0.001
Distance of donation to transplantation hospital (km)	110.0 (24.0, 261.0)	94.0 (16.0, 213.3)	<0.001	102.0 (20.0, 240.0)	166.0 (63.8, 339.3)	<0.001
DCD (%)	296 (7.2)	321 (12.2)	<0.001	501 (7.7)	116 (52.3)	<0.001
KDPI category (%)			0.140			0.060
<20%	1,602 (38.8)	979 (37.2)		2,514 (38.4)	67 (30.2)	
20%–34%	911 (22.1)	570 (21.7)		1,432 (21.9)	49 (22.1)	
35%–85%	1,542 (37.3)	1,018 (38.7)		2,459 (37.6)	101 (45.5)	
>85%	74 (1.8)	65 (2.5)		134 (2.0)	5 (2.3)	
CIT for kidney (hour)	9.77 (7.60,12.3)	19.3 (11.0, 26.7)	^a^	11.0 (8.20,18.2)	19.6 (15.5, 26.1)	^a^
CIT for liver (hour)	6.08 (5.00, 7.47)	5.90 (4.73,7.50)	^a^	5.98 (4.83,7.30)	13.0 (10.1,16.2)	^a^
Machine perfusion for liver (%)	57 (1.3)	165 (6.3)	<0.001	-	-	-
Machine perfusion for kidney (%)	-	-	-	2,467 (36.6)	165 (74.3)	<0.001
Outcomes						
DGF for kidney	1,256 (31.0)	662 (25.3)	<0.001	1857 (28.8)	61 (28.4)	0.898
PNF for kidney	285 (6.6)	194 (7.4)	0.213	461 (6.8)	18 (8.1)	0.465
PNF for liver	78 (1.8)	37 (1.4)	0.207	114 (1.6)	1 (0.5)	0.153
Re-transplantation (%)			0.576			0.235
Re-transplantation for kidney	15 (0.3)	11 (0.4)		26 (0.4)	0 (0.0)	
Re-transplantation for liver	44 (1.0)	29 (1.1)		68 (1.0)	5 (2.3)	
Re-transplantation for both organs	6 (0.1)	1 (0.0)		7 (0.1)	0 (0.0)	

Continuous data are presented as median (IQR): y.o., year old; %, percent; BMI, body mass index; NASH, nonalcoholic steatohepatitis; HCV, hepatitis C virus; MELD, model for end-stage liver disease; INR, international normalized ratio; PKD, polycystic kidney disease; DGF, delayed graft function; PNF, primary non-function; DCD, donation after circulatory death; KDPI, kidney donor profile index; CIT, cold ischemic time.

^a^ The comparison of CIT between MP and non-MP was not possible or did not reflect actual impact of CIT, since in MP cases, CIT recorded in the UNOS data included MP time.

Table 1 also shows the comparison of patients’ characteristics between liver MP group and no liver MP group. More than half of liver allografts treated with MP were DCD organs (52.3% vs. 7.7%, p < 0.001). Although the MELD score was lower in the liver MP group (median 27.0 vs. 29.0, p < 0.001), there were no statistically significant differences in the presence of dialysis or serum creatinine levels at the time of transplantation between two groups. The incidence of DGF was significantly lower in the kidney MP group compared to no kidney MP group. In contrast, there were no significant differences between MP and non-MP groups for either organ regarding the incidence of PNF (for kidney or liver) or the rate of re-transplantation.

### Kidney Machine Perfusion and 1-Year Kidney Graft Survival/1-Year Patient Survival

Cox hazard models were used to evaluate the factors related to 1-year kidney graft failure in kidney MP group and no kidney MP group (Supplementary Table S2; Table 2). KDPI >85% was an independent risk factor of 1-year kidney graft failure in the no kidney MP group [HR 2.03, 95% CI 1.20–3.44, p = 0.009]. However, when MP for kidney was used, KDPI >85% was not found to be the risk factor related to 1-year kidney graft failure [HR 1.41, 95% CI 0.79–2.52, p = 0.250]. There was no significant relationship between MP for the liver and 1-year kidney graft failure in either group. DCD was not identified as a risk factor for 1-year kidney graft failure in both groups (Table 2).

**TABLE 2:** Multivariable Cox proportional hazards model for 1-year kidney graft failure according to the presence of machine perfusion for kidney allograft.

Variables	No Kidney MP group		Kidney MP group	
	HR (95% CI)	*p*-value	HR (95% CI)	*p*-value
Recipient variables				
Age	1.02 (1.01–1.03)	0.002	1.01 (0.995–1.02)	0.238
Male	1.17 (0.96–1.42)	0.113	0.997 (0.80–1.25)	0.977
BMI	1.01 (0.99–1.02)	0.371	1.03 (1.01–1.05)	0.002
Race (ref: White)				
Black	0.80 (0.59–1.08)	0.143	1.28 (0.93–1.78)	0.132
Hispanic	0.84 (0.66–1.08)	0.173	0.89 (0.66–1.21)	0.461
Asian	0.90 (0.57–1.41)	0.635	1.00 (0.56–1.81)	0.990
Other	0.61 (0.25–1.49)	0.281	0.59 (0.22–1.60)	0.302
Diabetes	1.10 (0.90–1.33)	0.352	1.37 (1.09–1.73)	0.008
Donor variables				
Male	0.80 (0.66–0.96)	0.017	0.88 (0.70–1.10)	0.268
KDPI >85%	2.03 (1.20–3.44)	0.009	1.41 (0.79–2.52)	0.250
Race (ref: White)				
Black	0.82 (0.62–1.09)	0.169	1.20 (0.88–1.64)	0.255
Hispanic	0.99 (0.77–1.28)	0.933	1.15 (0.84–1.56)	0.383
Asian	1.65 (1.02–2.66)	0.042	1.17 (0.55–2.50)	0.684
Other	0.92 (0.41–2.07)	0.844	1.28 (0.53–3.13)	0.583
DCD	1.13 (0.80–1.60)	0.500	1.07 (0.75–1.54)	0.703
Machine perfusion for Liver	1.10 (0.90–1.33)	0.352	1.16 (0.69–1.94)	0.579

DCD, donation after circulatory death; KDPI, kidney donor profile index.

### Liver Machine Perfusion and 1-Year Liver Graft Survival/1-Year Patient Survival

We compared the risk factors related to 1-year liver graft failure in the liver MP group and the no liver MP group by using Cox hazard model (Supplementary Table S3; Table 3). DCD was found to be an independent risk factor of 1-year liver graft failure in the no liver MP group [HR 1.56, 95% CI 1.19–2.03, p = 0.001], but not in the liver MP group [HR 0.57, 95% CI 0.17–1.87, p = 0.353]. The MP for kidney was not related to 1-year liver graft survival in both groups (Table 3).

**TABLE 3:** Multivariable Cox proportional hazards model for 1-year liver graft failure according to the presence of machine perfusion for liver allograft.

Variables	No liver MP group		Liver MP group	
	HR (95% CI)	*p*-value	HR (95% CI)	*p*-value
Recipient variable				
Age	1.02 (1.01–1.03)	<0.001	0.98 (0.93–1.04)	0.544
Male	0.88 (0.75–1.04)	0.130	0.52 (0.16–1.66)	0.267
BMI	1.01 (0.99–1.02)	0.158	0.97 (0.88–1.07)	0.531
Race (ref: White)				
Black	1.14 (0.90–1.44)	0.278	1.21 (0.19–7.85)	0.843
Hispanic	0.85 (0.69–1.05)	0.136	0.42 (0.08–2.22)	0.306
Asian	0.97 (0.66–1.44)	0.894	-	0.987
Other	0.49 (0.22–1.11)	0.087	-	0.994
Diabetes	1.16 (0.98–1.36)	0.083	1.23 (0.44–3.47)	0.694
MELD score	1.02 (1.01–1.03)	<0.001	1.12 (1.02–1.23)	0.016
Donor variable				
Age	1.01 (0.99–1.01)	0.110	1.07 (1.01–1.14)	0.033
Male	0.88 (0.75–1.04)	0.130	0.75 (0.26–2.16)	0.595
BMI	1.00 (0.99–1.02)	0.509	0.99 (0.89–1.10)	0.803
Race (ref: White)				
Black	0.998 (0.79–1.25)	0.986	3.24 (0.57–18.4)	0.184
Hispanic	1.05 (0.84–1.30)	0.691	3.34 (0.66–17.0)	0.147
Asian	1.46 (0.94–2.28)	0.096	45.4 (3.41–602)	0.004
Other	0.81 (0.38–1.71)	0.579	2.91 (0.25–33.7)	0.392
Cause of death (ref: anoxia)				
cerebrovascular accident	1.16 (0.93–1.44)	0.184	0.08 (0.01–0.69)	0.022
trauma	1.06 (0.88–1.28)	0.565	0.45 (0.10–2.00)	0.291
DCD	1.56 (1.19–2.03)	0.001	0.57 (0.17–1.87)	0.353
Machine perfusion for kidney	1.12 (0.96–1.31)	0.159	1.07 (0.26–4.29)	0.928

MELD, model for end-stage liver disease; DCD, donation after circulatory death.

### The Influence of MP on DGF

Kidney MP was associated with decreased a risk of DGF among the recipients with KDPI >35%, as well as among the patients with KDPI >60%. [(OR 0.72, 95% CI 0.61–0.86, p < 0.001), (OR 0.74, 95% CI 0.56–0.98, p = 0.038), respectively] However, there was no significant association between kidney MP and DGF in the group with KDPI >85% (p = 0.182). Liver MP was not associated with DGF in all KDPI groups (Table 4).

**TABLE 4:** Multivariable logistic regression model for DGF.

Variables	KDPI >35		KDPI >60		KDPI >85	
	OR (95% CI)	*p*-value	OR (95% CI)	*p*-value	OR (95% CI)	*p*-value
Machine perfusion for kidney	0.72 (0.61–0.86)	<0.001	0.74 (0.56–0.98)	0.038	0.59 (0.27–1.28)	0.182
Machine perfusion for liver	0.71 (0.44–1.14)	0.154	0.52 (0.22–1.25)	0.144	1.05 (0.08–13.3)	0.968

Adjusted for recipient’s age, gender, BMI, race, diabetes mellitus, donor’s age, gender, BMI, race and DCD.

### The Influence of MP on 1-Year Graft Survival

To investigate the influence of MP for 1-year graft survival, we compared the 1-year graft survival between four groups; group 1: patients who did not use MP for both kidney and liver (N = 4,267), group 2: patients who used MP for only kidney (N = 2,467), group 3: patients who used MP for only liver (N = 57), group 4: patients who used MP for both kidney and liver (N = 165).

**FIGURE 3:**
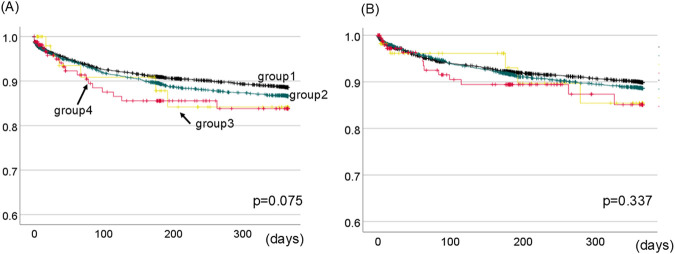
1-Year kidney **(A)** and liver **(B)** graft survival between four groups divided based on the presence of MP for kidney or liver. The black line shows group1 (patients who did not use MP for both kidney and liver). The green line shows group2 (patients who used MP for only kidney). The yellow line shows group3 (patients who used MP for only liver). The red line shows group4 (patients who used MP for both kidney and liver). There was no statistically difference in 1-year kidney or liver graft survival between four groups. [p = 0.075 **(A)**, p = 0.337 **(B)**, respectively.].

There was no statistically significant difference in 1-year kidney graft survival between the four groups (p = 0.075, Figure 3A). Similarly, we examined the influence of MP on 1-year liver graft survival. The 1-year liver graft survival was comparable between four groups (p = 0.337, Figure 3B).

## Discussion

The utilization of MP in SLKT has significantly increased in the U.S. The use of MP for kidney and liver increased from 21% to 51% and from 0% to 17%, respectively, between 2015 and 2023. Additionally, the proportion of SLKT from DCD rose from 4.5% in 2015 to 16% in 2023. Although MP has demonstrated many benefits in kidney-only and liver-only transplants [4, 26, 27], there are limited studies addressing the role of MP in SLKT. Of note, there are few studies that have examined the significance of MP in SLKT, particularly in the context of expanding donor pool. While DCD was a risk factor for 1-year liver graft failure in the absence of liver MP, it was not a risk factor when liver MP was used. Similarly, while KDPI >85% was associated with an increased risk of 1-year kidney graft failure without kidney MP, kidney MP might mitigate this risk. There was no difference in 1-year kidney or liver graft survival based on the use of MP for each organ individually or both, compared to no MP. Thus, MP for both organs might contribute to expanding the donor pool for SLKT without compromising post-transplant outcomes even with more use of marginal donor grafts.

The introduction of the MELD score into OPTN deceased donor liver allocation policy in 2002 has resulted in a substantial rise in SLKT in the US. [28]. Of the total number of SLKT from 2002 to 2012, 49% of donor kidneys had a KDPI <35% and were prioritized for pediatric candidates in kidney-alone allocation [28]. In 2017, an UNOS allocation policy for SLKT was established in the US, setting the eligibility criteria for SLKT. As a result, there was a temporary decline from 2016 to 2019 [29]. However, along with the increase of use of MP preservation in SLKT, the number of SLKT has shown a rising trend again since 2020. It should be noted that MP for kidney was used in approximately half of total SLKT in 2023, and MP for liver began being used in 16% from 2022, although MP for liver was rarely used until 2021. From these findings, it is indicated that MP has contributed to the increase in SLKT.

There are many studies which compared outcomes of DBD and DCD in SLKT. Croome et al compared outcomes of DCD SLKT performed between 2000 and 2010 and 2011–2018 by using the UNOS data. Improvement in patient, liver graft, and kidney graft survival rates in DCD-SLKT was seen between these two eras [30]. They concluded that patients who underwent DCD-SLKT achieved comparable outcomes to those of matched patients who underwent DBD-SLKT in recent periods. The effect of MP was not captured in their study. Vinson et al performed the study which compared the overall outcomes of accepting a DCD SLKT now vs. waiting for a DBD SLKT in patients waitlisted for SLKT, stratified by MELD score (≤20, 21–30, >30) [31]. DCD SLKT could be a preferred option for the patients with MELD score >30 (incremental value of 0.31 quality-adjusted life years for DCD vs. DBD) [31]. Our study, using more recent UNOS data, demonstrated that DCD was identified as the risk factor related to 1-year liver graft failure. Meanwhile, DCD was not associated with the outcome, even after adjusting for the MELD score, when MP for liver was available. Although the type of MP in this study cannot be distinguished, the MP for liver in SLKT has been increasing since 2018, and it is possible that NMP was predominantly used. NMP techniques focus on keeping the liver in a condition which is similar to physiological metabolism and supports its synthetic functions [3]. A randomized clinical trial in liver transplantation conducted in the United States demonstrated that NMP preservation of deceased donor livers significantly reduced the incidence of early allograft dysfunction and ischemic biliary complications [32]. The use of NMP also contributed to a significant increase in the utilization of DCD donors [32]. Therefore, it is indicated that the introduction of MP increased the feasibility of using DCD donors in SLKT as well. In this analysis, MP was not found to be a factor that improved liver graft survival, but further accumulation of cases may lead to positive expectations in the future.

As mentioned above, high-quality donor kidneys with lower KDPI have been often used for SLKT, which otherwise would have been allocated to the prioritized groups on the kidney transplant alone waiting list [33]. The KDPI of kidney grafts used for SLKT remains relatively low; however, the proportion of KDPI <20% decreased from 43.4% in 2015 to 36.0% in 2023, while the use of KDPI 35%–85% increased from 32.7% in 2015 to 39.9% in 2023. At present, though kidneys with KDPI >85% are infrequently used for SLK, to expand the donor pool, the use of higher KDPI organs is unavoidable. Montenovo et al investigated the effects of MP on development of DGF according to KDPI in kidney only transplantation. They found that MP was associated with significantly decreased development of DGF in donors with KDPI >60% [34]. In our study, MP for kidney was also associated with decreased a risk of DGF among the recipients with KDPI >60% as well as among the recipients with KDPI >35%. However, there was no significant association between MP for kidney and DGF in the group with KDPI >85%. The number of patients with KDPI >85% was 283, and 55 of them developed DGF. This relatively small sample size may have influenced the observed result. DGF has historically been associated with inferior graft survival [35]. Further research incorporating longer observation period could offer deeper insights into the impact of MP on long-term kidney graft survival.

Then, in which cases and how should MP techniques be applied? NMP offers preserving the organ by supplying oxygen under near-physiological conditions, which is associated with an increase in proteins that mediate the key metabolic processes, including fatty acid ß-oxidation, the tricarboxylic acid cycle, and acid phosphorylation [4]. NMP also enhances specific cellular defense mechanisms, producing an effect similar to ischemic preconditioning [4]. It has been reported that MP techniques were associated with lower rates of ischemic cholangiopathy in DCD liver transplantation due to the potential to reduce ischemic-reperfusion injury [32]. The use of MP for liver in DCD-SLKT may be recommended not only for expanding donor eligibility but also for reducing complications associated with DCD. As for the use of MP on the kidney, it might be better to use MP in cases with a higher KDPI. Bachmann et al demonstrated that KDPI correlated with glomerulosclerosis (r = 0.30), arteriosclerosis (r = 0.33), interstitial fibrosis, and tubular atrophy (r = 0.28) as well as the extent of acute tubular injury (r = 0.20) [36]. Acute tubular injury caused by longer ischemia time is the main cause of DGF [37]. In higher KDPI kidneys with significant pre-existing tubular atrophy or tubular injury, MP may be helpful in minimizing the additional impact of ischemia. Therefore, the indication of MP for both kidney and liver should be particularly considered in SLKT with DCD and/or higher KDPI.

There are some limitations in our study. First, the OPTN/UNOS registry lacks the detail of available MP devices. The pumping duration and additional MP characteristics such as HMP and NMP, as well as use of back-to-base MPs, were not available. NRP might have been used but there is not data for it in UNOS. Thus, we did not incorporate the CIT for each organ into the analysis, considering the CIT in cases with MP may not accurately reflect the actual time. It should be noted that comparison of CIT between MP and non-MP was not possible or did not reflect actual impact of CIT, since in MP cases, CIT recorded in the UNOS data included MP time. Additionally, there is no detailed data regarding organ procurement techniques in UNOS data. Second, this was a retrospective study using the OPTN/UNOS registry, which lacks donor and recipient clinical detail. We may not have sufficient data on the unknown factors or unmeasured confounding variables affecting graft survival.

There has been a rapid rise in the use of MP for both kidney and liver allograft in the US. Although DCD was a risk factor for liver graft failure without MP for liver, it ceased to be a risk factor when MP for liver was applied. Likewise, although KDPI >85% was linked to a higher risk of kidney graft failure without MP for kidney, MP for kidney might help reduce this risk. MP might also enable the use of lower-quality organs that are currently unsuitable for transplantation, thereby further expanding the donor pool in SLKT. Further investigations would be warranted to confirm these findings and the assessments of optimal candidates for maximizing the effectiveness of this valuable technology should be explored.

## Data Availability

The datasets presented in this study can be found in online repositories. The names of the repository/repositories and accession number(s) can be found in the article/Supplementary Material.

## Footnotes

1
https://srtr.transplant.hrsa.gov/ADR/Chapter?name=Preface&year=2023
