# Supplementary material for: The Individual Impact of Machine Perfusion on Liver and Kidney on Donor Expansion in Simultaneous Liver and Kidney Transplantation
Source: Transpl Int. 2025 Sep 9;38:14807. doi: 10.3389/ti.2025.14807 (PMC12454159; doi:10.3389/ti.2025.14807)
Supplement: Supplementary file 1 [file DataSheet1.pdf]

Supplementary Tables

Table S1. Background characteristics and graft outcomes.

Recipient Variables	N = 6956
Age (y.o.)	59.0 (50.0, 64.0)
Male (%)	4133 (59.4)
Race/ethnicity (%)	
White	4390 (63.1)
Black	840 (12.1)
Hispanic	1294 (18.6)
Asian	303 (4.4)
Other	129 (1.9)
BMI (kg/m^2)	27.5 (23.9, 32.1)
Diabetes (%)	2986 (43.0)
Etiology of liver disease (%)	
NASH	1637 (24.4)
Alcohol	1598 (23.8)
HCV	832 (12.4)
Biliary diseases	249 (3.7)
Other	2401 (35.7)
MELD score	29.0 (23.0, 35.0)
Encephalopathy (%)	
Grade I	2174 (32.2)
Grade II	3598 (53.4)
Grade III	971 (14.4)
Ascites (%)	3056 (45.3)
Albumin (g/dL)	3.20 (2.70, 3.70)
Sodium (mmol/L)	136.0 (133.0, 139.0)
INR	1.54 (1.20, 2.00)
Total bilirubin (g/dL)	2.40 (0.90, 6.80)
Etiology of kidney disease (%)	
Hepatorenal	2832 (40.7)
Diabetes	1361 (19.6)
PKD	401 (5.8)
Hypertension	348 (5.0)

Glomerulonephritis	268 (3.9)
Other	1746 (25.1)
Hemodialysis (%)	2119 (31.5)
Creatinine (mg/dL)	3.42 (2.28, 5.03)
eGFR (mL/min/1.73 m^2)	17.0 (11.0, 27.4)
DGF for kidney	1918 (27.6)
PNF for kidney	479 (6.9)
PNF for liver	115 (1.7)
Re-transplantation for kidney	26 (0.4)
Re-transplantation for liver	73 (1.0)
Re-transplantation for both organs	7 (0.1)
Donor Variables	
Age (y.o)	34.0 (25.0, 45.0)
Male (%)	2694 (38.7)
Race/ethnicity (%)	
White	4532 (65.2)
Black	1018 (14.6)
Hispanic	1144 (16.4)
Asian	165 (2.4)
Other	97 (1.4)
BMI (kg/m^2)	26.4 (23.1, 30.5)
Cause of death (%)	
anoxia	3030 (43.6)
cerebrovascular accident	1378 (19.8)
trauma	2331 (33.5)
Creatinine (mg/dL)	0.90 (0.70, 1.20)
Distance of donation to transplantation hospital (km)	104 (21.0, 244.0)
DCD (%)	617 (9.2)
KDPI category (%)	
<20%	2581 (38.2)
20-34%	1481 (21.9)
35-85%	2560 (36.8)
>85%	139 (2.0)
CIT for kidney (hour)	11.1 (8.28,18.8)
CIT for liver (hour)	6.00 (4.88, 7.48)

Machine perfusion for liver (%)	222 (3.2)
Machine perfusion for kidney (%)	2632 (37.8)
Outcomes	
DGF for kidney	1918 (27.6)
PNF for kidney	479 (6.9)
PNF for liver	115 (1.7)
Re-transplantation for kidney	26 (0.4)
Re-transplantation for liver	73 (1.0)
Re-transplantation for both organs	7 (0.1)

Continuous data are presented as median (IQR): y.o., year old; %, percent; BMI, body mass index; NASH, nonalcoholic steatohepatitis; HCV, hepatitis C virus; MELD, model for end-stage liver disease; INR, International Normalized Ratio; PKD, polycystic kidney disease; DGF, delayed graft function; PNF, primary non-function; DCD, donation after circulatory death; KDPI, Kidney Donor Profile Index; CIT, cold ischemic time

Table S2. Univariable Cox proportional hazards model for 1-year kidney graft failure in the group with or without machine perfusion for kidney.

	No Kidney MP group		Kidney MP group	
**Recipient Variables**	HR (95% CI)	*p*-value	HR (95% CI)	*p*-value
**Age**	1.02 (1.01-1.03)	0.002	1.01 (1.00-1.03)	0.013
**Male**	1.14 (0.94-1.38)	0.180	1.01 (0.81-1.26)	0.913
**Race/ethnicity (ref; White)**				
**Black**	0.76 (0.56- 1.03)	0.073	1.29 (0.94-1.77)	0.119
**Hispanic**	0.85 (0.66-1.08)	0.188	0.94 (0.69-1.27)	0.669
**Asian**	0.91 (0.59-1.42)	0.679	1.01 (0.57-1.81)	0.962
**Other**	0.56 (0.23-1.36)	0.200	0.64 (0.24-1.71)	0.370
**BMI**	0.998 (0.998-1.03)	0.769	1.00 (1.00-1.00)	0.019
**Diabetes**	1.17 (0.97-1.41)	0.096	1.50 (1.21-1.86)	<0.001
**Etiology of liver disease (ref; HCV)**				
**NASH**	1.43 (1.05-1.95)	0.025	1.51 (1.02-2.23)	0.039
**Alcohol**	1.03 (0.75-1.43)	0.847	0.82 (0.54-1.25)	0.355
**Biliary diseases**	0.99 (0.56-1.76)	0.979	1.34 (0.72-2.50)	0.362
**Other**	1.05 (0.77-1.43)	0.750	1.07 (0.72-1.58)	0.741
**MELD score**	1.01 (1.00-1.03)	0.039	1.01 (1.00-1.03)	0.084
**Encephalopathy (ref; Grade I)**				
**Grade II**	0.97 (0.79-1.20)	0.780	0.86 (0.67-1.09)	0.210
**Grade III**	1.37 (1.04-1.79)	0.024	1.15 (0.84-1.58)	0.379
**Ascites**	1.02 (0.85-1.23)	0.802	0.98 (0.79-1.22)	0.877
**Albumin**	0.88 (0.78-1.00)	0.048	0.93 (0.80-1.08)	0.345
**Sodium**	0.99 (0.97-1.01)	0.510	1.00 (0.98-1.03)	0.942
**INR**	0.99 (0.92-1.07)	0.875	1.03 (0.95-1.11)	0.485
**Total bilirubin**	1.01 (1.00-1.02)	0.037	1.01 (1.00-1.02)	0.028
**Etiology of kidney disease (ref; glomerulonephritis)**				
**Diabetes**	0.92 (0.57-1.49)	0.742	1.53 (0.81-2.86)	0.187
**Hypertension**	1.19 (0.69-2.06)	0.537	1.64 (0.79-3.42)	0.188
**PKD**	0.55 (0.28-1.08)	0.083	0.64 (0.29-1.43)	0.276
**Hepatorenal**	0.92 (0.58-1.46)	0.716	0.93 (0.50-1.73)	0.821

**Other**	0.95 (0.59-1.54)	0.844	1.19 (0.63-2.22)	0.597
**Hemodialysis**	1.69 (1.35-2.11)	<0.001	1.23 (0.97-1.58)	0.093
**Creatinine**	0.99 (0.95-1.03)	0.552	1.01 (0.97-1.06)	0.558
**eGFR**	1.00 (1.00-1.00)	<0.001	1.00 (1.00-1.01)	0.001
**Donor Variables**				
**Age**	1.02 (1.01-1.03)	<0.001	1.01 (1.00-1.02)	0.060
**Male**	0.77 (0.64-0.93)	0.006	0.90 (0.72-1.13)	0.365
**Race/ethnicity (ref; White)**				
**Black**	0.85 (0.65-1.13)	0.258	1.26 (0.93-1.70)	0.140
**Hispanic**	0.96 (0.74-1.23)	0.719	1.12 (0.83-1.52)	0.457
**Asian**	1.70 (1.06-2.74)	0.029	1.14 (0.54-2.42)	0.733
**Other**	0.915 (0.41-2.05)	0.830	1.25 (0.51-3.02)	0.628
**BMI**	1.01 (1.00-1.03)	<0.001	1.02 (1.00-1.03)	0.093
**Cause of death (ref: anoxia)**				
**cerebrovascular accident**	1.50 (1.20-1.88)	<0.001	1.48 (1.11-1.95)	0.007
**trauma**	0.93 (0.75-1.17)	0.537	1.02 (0.79-1.33)	0.874
**Creatinine**	1.01 (0.89-1.15)	0.883	1.07 (0.94-1.22)	0.283
**Distance of donation to transplantation hospital**	1.00(1.00-1.00)	0.271	1.00 (1.00-1.00)	0.188
**DCD**	1.16 (0.82-1.62)	0.407	1.06 (0.75-1.49)	0.756
**KDPI category (ref; <20%)**				
**20-34%**	0.92 (0.70-1.21)	0.531	0.94 (0.76-1.17)	0.412
**35-85%**	1.53 (1.24-1.89)	<0.001	1.22 (1.03-1.46)	0.023
**>85%**	2.38 (1.40-4.04)	0.001	1.36 (0.82-2.25)	0.053
**Machine perfusion for liver**	1.31 (0.59-2.94)	0.508	1.26 (0.79-2.03)	0.336

NASH, nonalcoholic steatohepatitis; HCV, hepatitis C virus; MELD, model for end-stage liver disease; INR, International Normalized Ratio; PKD, polycystic kidney disease; DCD, donation after circulatory death, KDPI, Kidney Donor Profile Index

Table S3. Univariable Cox proportional hazards model for 1-year liver graft failure in the group with or without machine perfusion for liver.

	No liver MP group		Liver MP group	
**Recipient Variables**	HR (95% CI)	*p*-value	HR (95% CI)	*p*-value
**Age**	1.02 (1.01-1.03)	<0.001	0.98 (0.94-1.02)	0.376
**Male**	1.09 (0.93-1.28)	0.279	0.62 (0.25-1.52)	0.292
**Race/ethnicity (ref; White)**				
**Black**	1.06 (0.84-1.33)	0.633	1.55 (0.44-5.39)	0.494
**Hispanic**	0.89 (0.73-1.10)	0.293	0.44 (0.10-1.95)	0.282
**Asian**	0.96 (0.65-1.42)	0.845	-	0.983
**Other**	0.49 (0.22-1.09)	0.081	-	0.991
**BMI**	1.00 (1.00-1.00)	0.508	1.02 (0.95-1.08)	0.613
**Diabetes**	1.27 (1.09-1.48)	0.002	1.06 (0.43-2.61)	0.896
**Etiology of liver disease (ref; HCV)**				
**NASH**	1.52 (1.17-1.99)	0.002	0.54 (0.11-2.81)	0.466
**Alcohol**	0.89 (0.67-1.18)	0.413	0.21 (0.02-2.37)	0.209
**Biliary diseases**	1.27 (0.81-1.99)	0.291	1.08 (0.10-11.99)	0.947
**Other**	1.14 (0.88-1.49)	0.320	0.69 (0.15-3.17)	0.638
**MELD score**	1.01 (1.00-1.02)	0.018	1.12 (1.06-1.19)	<0.001
**Encephalopathy (ref; Grade I)**				
**Grade II**	0.81 (0.68-0.96)	0.015	1.49 (0.41-5.41)	0.546
**Grade III**	1.20 (0.96-1.50)	0.110	2.79 (0.70-11.17)	0.147
**Ascites**	1.05 (0.90-1.22)	0.568	0.56 (0.21-1.48)	0.242
**Albumin**	0.89 (0.80-0.99)	0.034	0.68 (0.37-1.28)	0.235
**Sodium**	1.00 (0.98-1.01)	0.526	0.95 (0.86-1.06)	0.369
**INR**	1.01 (0.96-1.06)	0.692	2.04 (1.13-3.66)	0.017
**Total bilirubin**	1.01 (1.00-1.02)	0.001	1.05 (1.02-1.08)	0.001
**Etiology of kidney disease (ref; Glomerulonephritis)**				
**Diabetes**	0.95 (0.64-1.41)	0.796	-	0.916
**Hypertension**	1.14 (0.72-1.80)	0.573	-	0.918
**PKD**	0.52 (0.31-0.90)	0.018	-	1.000

**Hepatorenal**	0.76 (0.52-1.10)	0.147	-	0.922
**Other**	0.89 (0.60-1.31)	0.555	-	0.919
**Hemodialysis**	1.41 (1.18-1.68)	<0.001	1.09 (0.43-2.77)	0.859
**Creatinine**	0.99 (0.95-1.02)	0.428	0.77 (0.58-1.01)	0.061
**eGFR**	1.00 (1.00-1.00)	<0.001	1.01 (1.01-1.02)	<0.001
**Donor Variables**				
**Age**	1.01 (1.00-1.01)	0.011	1.02 (0.98-1.07)	0.258
**Male**	0.86 (0.73-1.00)	0.051	0.54 (0.22-1.33)	0.181
**Race/ethnicity (ref; White)**				
**Black**	0.97 (0.77-1.22)	0.970	2.66 (0.73-9.68)	0.139
**Hispanic**	1.04 (0.84-1.28)	0.745	1.77 (0.49-6.43)	0.388
**Asian**	1.52 (0.98-2.36)	0.060	12.0 (2.52-57.3)	0.002
**Other**	0.77 (0.36-1.62)	0.489	4.84 (0.62-37.9)	0.133
**BMI**	1.01 (1.00-1.02)	0.203	0.99 (0.92-1.07)	0.842
**Cause of death (ref: anoxia)**				
**cerebrovascular accident**	1.27 (1.04-1.55)	0.020	0.59 (0.17-2.10)	0.592
**trauma**	1.03 (0.85-1.23)	0.789	0.43 (0.12-1.54)	0.434
**Creatinine**	1.04 (0.94-1.15)	0.489	1.35 (0.76-2.41)	0.312
**Distance of donation to transplantation hospital**	1.00 (1.00-1.00)	0.273	1.00 (1.00-1.00)	0.422
**DCD**	1.41 (1.09-1.83)	0.009	0.38 (0.14-1.06)	0.065
**KDPI category (ref; <20%)**				
**20-34%**	0.94 (0.76-1.17)	0.586	0.66 (0.17-2.64)	0.557
**35-85%**	1.22 (1.03-1.46)	0.025	1.08 (0.38-3.04)	0.883
**>85%**	1.36 (0.82-2.25)	0.237	2.68 (0.32-22.36)	0.362
**Machine perfusion for kidney**	1.12 (0.96-1.31)	0.159	1.13 (0.41-3.14)	0.814

NASH, nonalcoholic steatohepatitis; HCV, hepatitis C virus; MELD, model for end-stage liver disease; INR, International Normalized Ratio; PKD, polycystic kidney disease; DCD, donation after circulatory death, KDPI, Kidney Donor Profile Index
